# High ambient temperature effects on the anthropometric status of the population: A systematic review and meta-analysis

**DOI:** 10.1371/journal.pone.0344186

**Published:** 2026-04-01

**Authors:** Priscila Ribas de Farias Costa, Rita de Cássia Ribeiro-Silva, Rafaela de Oliveira Santos, Karine Brito Beck da S. Magalhães, Ellayne Souza Cerqueira, Aline dos Santos Rocha, Lisianne Passos Luz, Lais Helena Leandro Ribeiro, Otavio T. Ranzani, Ismael Silveira, Maxine Pepper, Enny S. Paixao, Maurício Lima Barreto

**Affiliations:** 1 Post-Graduation Program in Food, Nutrition and Health, School of Nutrition, Federal University of Bahia, Salvador, Brazil; 2 Center for Data and Knowledge Integration for Health (CIDACS), Oswaldo Cruz Foundation, Salvador, Brazil; 3 Institute of Collective Health, Federal University of Bahia, Salvador, Brazil; 4 Institut de Recerca Sant Pau (IR SANTPAU), ISGlobal, Barcelona, Spain; 5 Department of Epidemiology and Population Health, London School of Hygiene and Tropical Medicine, London, United Kingdom; University of Michigan, UNITED STATES OF AMERICA

## Abstract

The effects of high ambient temperatures and heatwaves on health outcomes are well established, yet their impact on nutritional status remains poorly understood. This systematic review and meta-analysis aimed to synthesize current evidence on the association between elevated temperatures and anthropometric indicators in the general population. The protocol was registered in PROSPERO (CRD42024555573), and a systematic search of electronic databases was conducted in September 2025 using terms related to “high temperatures,” “heatwaves,” and “anthropometric indicators.” Twenty studies were included, comprising 2,943,695 participants, predominantly children under five years of age, mainly from Sub-Saharan Africa. Studies demonstrated methodological heterogeneity, with mixed findings for height-for-age (HAZ) and weight-for-age (WAZ), but generally indicated an inverse relationship between high temperatures and anthropometric outcomes. In adults, elevated temperatures were associated with both underweight and obesity, highlighting complex effects on nutritional status. Meta-analyses in children revealed reductions of 0.12 SD in Weight-for-Height Z-scores and 0.03 SD in HAZ per 1°C increase in average temperature. While effect sizes at the individual level appear to be modest, the population-level implications could be considerable given the widespread and increasing exposure to heat. These findings suggest a potential link between thermal stress and nutritional status, underscoring the need for longitudinal and geographically diverse studies to further clarify causal pathways, identify vulnerable groups, and inform evidence-based climate-adaptive public health strategies aimed at mitigating the potential nutritional consequences of rising temperatures.

## Introduction

Malnutrition, which encompasses both undernutrition (underweight, wasting, stunting, and weight loss) and overnutrition (overweight and obesity), poses a significant challenge to the health of individuals and populations. It creates a heavy burden for healthcare systems and adversely impacts economic productivity. [1]. The human and socioeconomic costs of malnutrition are enormous, disproportionately affecting the poorest, particularly women, children, and the elderly. The harmful effects of malnutrition can impact an individual’s health throughout their life, beginning early and persisting into old age. It affects physical, mental, and social well-being, while also increasing the risks of morbidity and mortality [2,3].

Emerging research on climate and health suggests a link between high temperatures and malnutrition [2,4,5]. The pathways via which high temperatures impact health and nutrition are complex, involving short-, medium-, and long-term mechanisms, and may vary by geography, socioeconomic context, and ecosystem [6]. Children are particularly vulnerable to the harmful effects of extreme heat due to their developing thermoregulatory systems, which are less efficient than those of adults [7]. This vulnerability is further exacerbated by prolonged outdoor exposure during play and the environmental conditions of households situated in agricultural regions, where extreme heat may be more intense and shelter less accessible. Heat stress can cause a series of acute health problems, including loss of appetite, poor nutrient retention, and increased diarrhoea and dehydration, hence leading to poor nutrient and calorie absorption and ultimately weight loss [8].

Furthermore, high temperatures can reduce crop yields threatening household food security, and increase water scarcity which contributes to poor sanitation [9,10]. They also alter the transmission dynamics of infectious diseases [11]. In addition, they increase the risk of violent conflicts [12], reduce work productivity, income, and economic growth [13]. These indirect effects of high temperature can, in turn, influence nutritional outcomes among adults and the elderly, although these relationships continue to be poorly understood [14]. High temperatures have also been suggested to alter physical activity levels, favouring a positive calorie balance, thereby directly impacting adult obesity [15–18]. Many of these adverse health consequences are concentrated in the populations of low- and middle-income countries who are exposed to higher average temperatures, may have a lower adaptive capacity, and where the means of subsistence are more directly dependent on environmental conditions.

In recent years, an increasing number of studies suggested an association between high temperatures and nutritional outcomes. However, this evidence has not yet been systematically compiled and evaluated. Therefore, this systematic review aims to synthesise previous research on the association between high ambient temperatures and heatwaves and indicators of nutritional status (anthropometric indicators) in all age groups. By mapping the global literature, our review can inform the development of climate adaptation policies which mitigate the risk of malnutrition associated with extreme heat.

## Methods

This is a systematic review and meta-analysis which evaluated the evidence available on the association between extreme heat and heatwaves and anthropometric indicators in the general population, whose protocol was submitted to PROSPERO (International prospective register of systematic reviews) and registered under number CRD42024555573. We adopted the methodological recommendations proposed by Cochrane [19] and the wording proposed by PRISMA [20] (S1 Table).

### Inclusion and exclusion criteria

The inclusion and exclusion criteria were defined in accordance with the PECOS acronym (Table 1). Studies included in the systematic review must meet the following criteria: (1) population: no age or physiological restrictions were applied, encompassing the general population across the life course (children, adolescents, adults, pregnant women, and the elderly); (2) Exposure: ambient temperature metrics or heatwave episodes, including both station-based and satellite-derived measurements; (3) Comparator: lower temperature levels, non-heatwave periods, or reference temperature ranges defined within each primary study; (4) Outcomes: objectively measured anthropometric indicators, analyzed as either continuous or categorical variables. These included standardized Z-scores (WAZ, HAZ, WHZ, BAZ), BMI, waist circumference, and body composition metrics (percentage of body fat and lean mass); (5) Study design: observational studies (cohort, cross-sectional, and case-control) were prioritized to capture real-world environmental impacts. To ensure quantitative robustness, we only included studies reporting at least one effect measure with its respective precision estimate – specifically, Relative Risk (RR), Prevalence Ratio (PR), or Odds Ratio (OR) with 95% Confidence Intervals (CI) for categorical data; or means, standard deviations, β coefficients, or correlation coefficients with associated p-values for continuous data.

**Table 1:** PECOS criteria for study selection.

Parameters	Criteria
Population	General (children, adolescents, pregnant women, adults and elderly people)
Exposure	High temperatures, extreme heat, heatwaves, and high ambient temperature (according to the classification adopted by the authors)
Comparator	Population exposed to lower temperature levels or not exposed to heatwaves
Outcomes	Anthropometric indicators
Setting or study design	Observational studies, such as cross-sectional, panel, cohort, and case-control studies

The following were adopted as exclusion criteria: (1) study population representing a select group of individuals with chronic or high risk diseases, or users of medications which may alter anthropometric measurements or pressure levels, such as nephropathies, neoplasias, chronic liver diseases, lupus, Crohn’s disease, mental illnesses, HIV, and Down’s syndrome, among others; (2) review studies, ecological studies and/or case reports; (3) studies which did not evaluate the outcomes covered in this research; and (4) studies which did not use temperature, or heatwaves, as an exposure variable.

### Search strategy

A comprehensive systematic search was conducted across the following electronic databases: PubMed/MEDLINE, EMBASE, Web of Science, and the Virtual Health Library (BVS), which encompasses LILACS, IBECS, WHO IRIS, CUMED, BDENF, PAHO, VENTIDEX, ARGMSAL, BINACS, and LIPECS. To minimize publication bias, Google Scholar was used to retrieve grey literature published up to October 13, 2025. No restrictions were imposed regarding date, language, or search filters to ensure maximum sensitivity. The strategy was developed using a combination of controlled vocabulary – specifically Medical Subject Headings (MeSH) for PubMed/BVS, Embase Subject Headings (Emtree), and Health Science Descriptors (DeCS) for LILACS – supplemented by a wide range of entry terms and non-controlled vocabulary (keywords) to enhance the retrieval of unindexed or recently published studies. The exposure and outcome terms and their respective synonyms were used in the search strategy, with the aim of including all studies relevant to this topic. The search strings were constructed using Boolean operators “OR” to group synonyms for exposure and outcome, and “AND” to intersect these core concepts [19].

We selected the Pubmed/MEDLINE MeSH (Medical Subject Headings) database descriptors. We also opted for sensitivity, with the inclusion of entry terms and non-controlled vocabulary. We developed Boolean combinations of words (separated by outcome) for database searches using MeSH descriptors in PUBMED/MEDLINE, BVS, and Web of Science, and also on Google Scholar. Regarding the LILACS database search, we used selected Virtual Health Library (Biblioteca Virtual em Saúde – BVC) DeCS (Health Science Descriptors) and also developed Boolean expressions of words for this search.

Lastly, we used Embase EMTREE (Embase Subject Headings) controlled vocabulary descriptors to construct Boolean expressions of words to search for articles indexed in this database. We also conducted a manual search of the reference lists of the studies included in the review, and relevant reviews identified during the selection process, in order to retrieve those which had not been retained by database searches.

The high sensitivity of the final search strategy was validated through a sentinel-based verification test; four pre-selected primary studies (sentinels) meeting all eligibility criteria were used as benchmarks, and the strategy successfully retrieved 100% of these articles. A detailed description of the full search strings for each database is provided in the Supporting Information (S2 Table).

### Selection of articles

We exported the citations from each database to the Covidence review software, Veritas Health Innovation, Melbourne, Australia (available at www.covidence.org), where any duplicates were removed. We executed a pilot test in the initial study selection stage, with the aim of “calibrating” the reviewers’ decisions and, if necessary, improving the clarity of the eligibility criteria. For the test, ten records identified by the search strategy were randomly selected, with two reviewers assessing the titles/abstract using eligibility criteria. The reviewers achieved an agreement rate higher than 75% in the pilot test, indicating the reviewers’ understanding of the eligibility criteria.

Following the pilot test, we screened the titles/abstracts (stage I) of all the sources retrieved in the search, following the eligibility criteria. In stage II of the selection, we reviewed the full text of the records selected in stage I, and of those whose eligibility was still uncertain. Two reviewers worked independently, and any inconsistencies in classifying the decisions were discussed with a third reviewer.

In stage II, the excluded sources were recorded on Covidence, along with the reason for their exclusion, and the entire study selection process was detailed in a PRISMA flowchart. The research team decided which studies should be included in the final selection for data summary.

### Data extraction and quality

Two independent reviewers systematically executed the data extraction, and any divergences were resolved through discussion with a third reviewer.

Following the final article selection, the data were extracted on a form using COVIDENCE software. In order to increase consistency among reviewers, and guarantee validity, we conducted another pilot test of the data extraction form in a random sample of five studies, and a third reviewer confirmed content accuracy. Following the pilot test, information was extracted from all the included studies, which included: the first author; study design and location, which was described in accordance with the country of the population analyzed; year of publication; follow-up period, when applicable; the study population was characterized according to the sample size, sex, age range, and/or average age of participants, and recruitment method; definition of the exposition: extreme temperature or heatwave classification method; exposure data source; year of data collection; statistical approach used, in addition to specifications in relation to instruments, indicators, and methods to identify the outcome; outcome evaluated: anthropometric indicators (WAZ, HAZ, WHZ, BMI, WC, arm circumference); main results found: measures of association (OR, RR, differences in the average outcome values in the exposed and non-exposed groups, and the linear regression β coefficient, or correlation coefficient); and study limitations. The authors of the selected studies were contacted by email to provide incomplete data or for any clarification as to the metrics evaluated that were incomplete or missing.

The risk of bias evaluation for each study included in the systematic review was conducted using the tool developed by the Office of Health Assessment and Translation (OHAT) [21], and is designed specifically for environmental health research [22]. Cohort and cross-sectional/panel studies were evaluated based on five categories (selection, confounding, exclusion/attrition, detection, selective reporting, and other sources of bias) which included seven questions (three classified as key criteria, and four as other criteria), with the following response options for each question: 1) definitively low risk of bias, 2) probably low risk of bias, 3) probably high risk of bias, and 4) definitively high risk of bias^22^. Two evaluators independently classified the studies, and then a consensus decision was achieved through discussion. Applying OHAT guidance, the publications were classified as Tier 1 (evaluated as ‘definitively’ or ‘probably low’ risk of bias in the three key criteria, and as ‘definitively’ or ‘probably low’ in the majority of the other criteria); Tier 3 (evaluated as ‘definitively’ or ‘probably high’ risk of bias in the three key criteria, and as ‘definitively’ or ‘probably high’ risk of bias in the majority of the other criteria); and Tier 2 (when the study does not meet the criteria for Tier 1 or Tier 3).

### Meta-analysis

For all studies included in this review, a narrative data summary was presented. For studies considered combinable [19], a quantitative data summary was conducted using meta-analysis. Due to the substantial heterogeneity in exposure metrics and life-course physiological responses identified during the systematic review, the quantitative synthesis (meta-analysis) was strategically restricted to studies involving pediatric populations. This subset demonstrated sufficient methodological homogeneity for statistical pooling, specifically concerning exposure definitions – limited to monthly or annual mean temperatures and maximum mean temperatures – and standardized nutritional outcomes (WAZ, HAZ, and WHZ) (S3 File). By applying these stringent comparability criteria, we aimed to minimize biases and ensure a robust estimation of the pooled effect sizes within this high-vulnerability group. No combinable studies were identified for other age groups or anthropometric indicators.

The extent of the heterogeneity in the meta-analysis heterogeneity was tested using Cochran’s Q test and quantified by the inconsistency test (statistic I^2^). This statistic determines the magnitude of the heterogeneity by the proportion of the total variation between studies, due to heterogeneity [19,23]. The p-value is frequently cited as an indication of the extent of variability in studies. Thus, we used the chi-squared test to evaluate the significance of the heterogeneity. Therefore, we adopted a p-value of <0.05 as the significance level, with the aim of detecting the heterogeneity of the study results [19,23].

We used the *metan* command for the meta-analyses, with the specification of two variables, assuming this to be the measure of effect (beta coefficient), and its respective standard errors. Given the small number of available studies for certain indicators, which resulted in non-normal distributions of effect sizes, continuous beta coefficients and their standard errors were log-transformed to stabilize variances and normalize the distribution prior to pooling. Following the meta-analysis, the eform option was applied to back-transform the pooled estimates to their original linear scale, ensuring the results are presented in an interpretable form. The summary effect was calculated using random-effect models with the restricted maximum likelihood (REML) method [19,23].

Considering the limited number of studies included in the meta-analyses, we were not able to investigate the causes of heterogeneity in the studies, whether by subgroup analysis or meta-regression; nor was an analysis of the publication bias conducted through the funnel chart and Egger’s test [19,23].

The statistical analysis was conducted using STATA for MAC statistics software (Version 16.0, Stata Corp LP, College Station, Texas).

The Grading of Recommendations Assessment, Development and Evaluation (GRADE) approach was used to rate the certainty of the evidence [24]. A Summary of Findings (SoF) table was prepared using the GRADEpro online software (GRADE Working Group, McMaster University) [25].

### Ethical approval

Ethical approval was not required since this study does not involve human participants.

## Results

### Study and population characteristics

**Fig 1:**
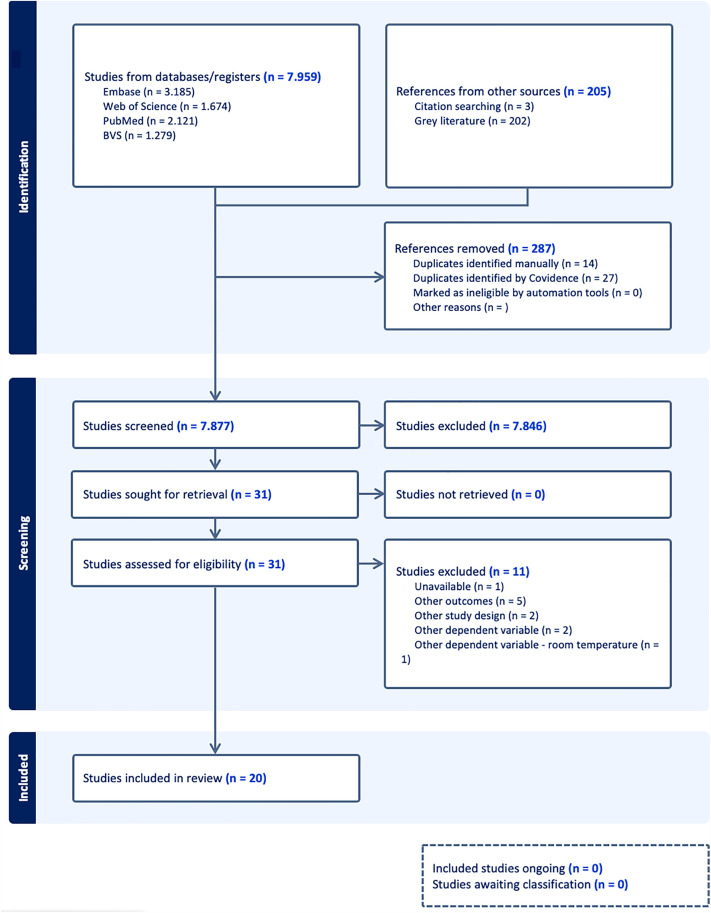
PRISMA flowchart describing the study selection process.

The database search identified 7,959 published articles and 205 documents were captured through citation searching and grey literature, totalling 8,164 documents published between 2012 and 2025. After removing 287 duplicates, 7,877 titles and abstracts were screened, with 31 articles read in full. A total of 20 studies were included in the systematic review (Fig 1).

Of the 20 studies, 16 evaluated the effects of temperature on anthropometric indicators of children under the age of 5 (n = 2,724,810) [4,5,8,26–38]; two on adult and elderly people (n = 205,789) [14,39]; one on adolescents and adults (n = 12,509) [40]; and one on the elderly (n = 587) [41], totalling 2,943,695 individuals. With the exception of the study conducted by Wallwork et al. (2017) [41], all studies used secondary data from demographic and health surveys (DHS) (Table 2).

**Table 2:** Summary of study characteristics included in the systematic review.

Author	Country	Study Design	Age	Sample Size	Outcomes	Exposure (Temperature)	Exposure Window	Results
Amondo, Nshakira‑Rukundo & Mirzabaev, 2023 [26]	Uganda	Panel study	Children aged between 6 and 59 months	1,397	HAZ	Heatwave (monthly temperatures above 29°C (84.2°F).	Last five years	**HAZ**: a heatwave in the main season and the last five years reduced calories, protein, zinc and vitamin A supply. A 10% decrease in zinc supply decreased HAZ by approximately 0.139–0.164 SD, and increased the probability of stunting, ranging from 3.1 to 3.5 percentage points.
Ampofo, Churchill & Churchill, 2025 [39]	Australia	Cohort Study	Adults and older people	184,799	BMI (self-reported)	Days exposed to temperature above 30ºC	16 years	**BMI:** an additional day of exposure to temperatures above 30◦C in a year is associated with a 0.02 percent increase in BMI. **Obesity:** an additional day of exposure to average temperatures above 30◦*C* is associated with a 0.2 percent increase in the probability of being obese.
Anttila-Hughes, Jina & McCord, 2021 [27]	51 countries (Not informed)	Panel study	Children aged under 4	1,253,176	WAZ WHZ BAZ Underweight Wasting	NINO 3.4 index of equatorial Pacific Sea surface temperature.	From May of one year to April of the next year	**WAZ**: a 1°C increase in the ENSO index is associated with 0.03σ (p = 0.02) average decrease in WAZ. **Wasting**: the risk of wasting is similarly positive, but not significant (0.3 p.p./°C, p = 0.21). **Underweight**: warmer ENSO increases the prevalence of being significantly underweight, by 0.6 percentage points per 1°C (p < 0.05).
Baker & Anttila-Hughes, 2020 [8]	30 Sub-Saharan African countries	Panel study	Children aged between 1 and 5	190,000	WHZ HAZ WAZ	Average monthly temperature	Annual and lifetime period	**WHZ**: a 1ºC change in annual temperature leads to an approximate 0.08σ decline in WHZ (adding temperature effects across 12 months in the year). A lifetime average temperature from 25 to 30ºC is associated with an approximate 0.5σ decrease in WHZ. **HAZ**: effect of temperatures not found. **WAZ**: effect of temperatures not found.
Blom, Ortiz-Bobea & Hoddinott, 2022 [28]	Benin, Burkina Faso, the Ivory Coast, Ghana, and Togo	Panel study	Children aged between 3 and 36 months	32,036	HAZ WHZ	Average hours per month over the exposure window bins: ≤ 25ºC, 25–30ºC, 30–35ºC, and >35ºC	HAZ: lifetime exposure WHZ: 90 days prior to the interview date	**HAZ**: exposure to temperatures above 35ºC decreases HAZ, and increases the risk of stunting. HAZ decrease of 18% for each 100 h of exposure above 35ºC. **WHZ**: decrease by 0.10 SD per 100h increase in average monthly exposure to temperatures between 30–35ºC
Davenport et al., 2017 [29]	13 Sub-Saharan African countries	Panel study	Children aged under 5	60,577	HAZ	Number of days where the maximum daytime temperature exceeds 37.7°C	One year before birth, until the interview date	**HAZ**: negative effect of heating on the child growth deficit, with the decrease of a −0.01 standard deviation in HAZ, while not statistically significant.
Grace et al., 2012 [30]	Kenya	Cross-sectional study	Children aged between 1 and 5	2,255	HAZ	Average temperature over the growing season, and the average of these values over the child’s life	Lifetime period	**HAZ**: temperature appears to have no significant impact on HAZ [ß = −0.0385; p > 0.05]
Ahmed Hanifi, Menon & Quisumbing, 2022 [32]	Bangladesh	Panel study	Children aged between 0 and 3	19,357	Upper arm circumference (MUAC)	- Monthly average temperature - Three temperature bins: 15–20ºC, 25–30ºC, and >30ºC	Month of conception In utero (by trimester) Month and year of birth Lifetime	**MUAC**: we found that temperatures that exceed 25ºC in the month and year of birth decrease the mid upper arm circumference (MUAC) by 1.476 cm (p < 0.01), and an over 30ºC decrease in MUAC to 2.115 cm (p < 0.05).
Islam et al., 2025 [31]	Pakistan	Cross-sectional study	Children aged under 2	29,887	WHZ HAZ	Annual averages temperatures Changes in the average temperatures between 2 periods [average (1981–2000) – annual average (2007–2017)]	Lifetime	**HAZ:** 1ºC increase in temperature was associated with a reduction in HAZ score in children under 24 mo (β −0.24, 95% CI: -0.41, –0.06) **WHZ:** 1ºC increase in temperature was associated with a reduction in WHZ score in children under 24 mo (β −0.33, 95% CI: -0.49, –0.17, P < 0.001).
Kamiya, Kishida & Tanou, 2025 [37]	Mali	Cross-sectional study	Children aged under 5	12,281	HAZ (stunting) WAZ (underweight) WHZ (wasting)	- Average temperature (3, 6, 12 and 24 months)	- Three-month, six-month, one-year and two-year periods preceding the survey	**Stunting:** in the north, an increase in average temperature (°C) over the last 6 months was significantly associated with higher odds of stunting (OR = 1.52; p = 0.028); also, an increase in average temperature over the last year was significantly associated with higher odds of stunting (OR = 1.351, p = 0.036). Similarly, an increase in average temperature over the last two years was associated with higher odds of stunting (OR = 1.400, p = 0.032). Conversely, in the south, an increase in average temperature over the last year (OR = 0.853, p = 0.012) and two years (OR = 0.832, p = 0.003) was associated with lower odds of stunting. **Underweight:** an increase in average temperature over the last year was significantly associated with higher odds of underweight (OR = 1.565, p = 0.001); an increase in average temperature over the last two years was associated with higher odds of underweight (OR = 1.598; p = 0.002). **Wasting:** in the north, an increase in average temperature over the last year (OR = 5.668, p = 0.0001) and two years (OR = 2.654, p = 0.024) was significantly associated with higher odds of wasting.
Kanazawa, 2020 [40]	USA	Cohort study	Adolescents and adults	12,509	BMI Overweight Obesity	- Average number of annual days with temperature higher than 90°F (32.2ºC) - Average maximum daily temperature - Average minimum daily temperature - Average total annual hours of sunshine	Not clear (one year before interview?)	**BMI**: high temperatures were significantly associated with a higher BMI, weight, being overweight, and obesity. They found that the daily maximum temperature was positively associated with BMI (β = 0.036; p < 0.001), measured weight (β = 0.098; p < 0.001), being **overweight** (β = 0.008; p < 0.001), and **obesity** (β = 0.011; p < 0.001).
McMahon & Gray, 2021 [33]	Bangladesh, India, Nepal, and Pakistan	Panel study	Children aged between 2 and 5	222,572	HAZ	- Monthly climate anomalies (using the historical average and historical standard deviation in each province) − 9-month anomalies − 12-month anomalies for the first (0–11 months) and second (12–23) year of life	During the prenatal period and first 2 years of life	**HAZ**: One additional unit of heat decreases the likelihood of stunting by 3.4% (p = 0.078), and increases HAZ by 2.7% (p = 0.055) when the anomaly occurs during the first and second years, respectively.
Merwe, Clance & Yitbarek, 2022 [34]	Nigeria	Cohort study	Children under the age of 5	3,511	HAZ WAZ	Monthly average temperature Monthly maximum average temperature	From July (the year prior to the survey) to June, (year of the survey)	**HAZ**: a one-unit (◦C) increase in temperature increases the probability of child stunting by between 18.6% and 22.3% **WAZ**: the probability of a child being underweight increases by between 7.9% and 15.2% with a one-unit (◦C) increase in temperature
Mueller & Gray, 2018 [14]	China	Cohort study	Adults and older people	20,990	BMI	Temperature anomalies (measured as standardized anomalies, or z-scores, defined as the temperature deviation during the calendar year of interview from the 1981–2010 average temperature, divided by the standard deviation in the temperature measured over the same period)	Year of the interview	**BMI**: temperature anomalies increase the probability of being underweight in individuals aged between 41 and 60 (F statistic = 0.006; p = 0.003), and more significantly in those aged over 60 (F statistic = 0.011; p = 0.004). Extremely dry and hot conditions lead to a 3.3 percentage point increase in the underweight status for the group of those aged over 60.
Randell, Gray & Grace, 2020 [4]	Ethiopia	Panel study	Children aged between 1 and 5	23,026	HAZ	Average maximum daily temperature (°C)	Child’s prenatal period and early life (birth and through the time of the survey)	**HAZ**: 1°C increase in average prenatal temperature is associated with a 16% (p < 0.01) and 28% (p < 0.001) increase in the odds of stunting and severe stunting, respectively. And 1°C increase in average early life temperature is associated with a 13% (p < 0.01) and 23% (p < 0.001) increase in the odds of stunting and severe stunting, respectively.
Raza et al., 2025 [38]	Bangladesh	Panel study	Children aged between 2 and 5	24,035	HAZ (Stunting)	Extreme heat (defined as the number of days when the maximum temperature exceeded the 80th percentile, based on a 10-year, month-on-month reference period for the warm months (March–November).	Child’s total exposure to extreme heat in utero, 0–23 months of age, and pooled to reflect the first 1000 days.	**Stunting:** a 1% increase in extreme heat days during the first 1000 days of life was associated with higher odds of stunting (OR = 1.56, 95%CI: 1.25–1.95, p < 0.0001) at 24–59 months of age. Post-birth exposure to extreme heat showed a stronger association with stunting (OR 1.67, 95%CI: 1.37–2.03, p = 0.063) than in utero exposure (OR 1.28, 95%CI: 1.14–1.44, p < 0.0001).
Rojas, Gray & West, 2023 [35]	Burkina Faso	Panel study	Children aged between 2 and 5	12,321	HAZ	Temperature anomalies (daily average temperature higher than the 95th percentile)	Prenatal period, the first and second year of life	**HAZ**: temperature anomalies in the first year were positively associated with HAZ (ß = 0.066, p < 0.01). Temperature anomalies in the prenatal period and second year of life were not associated with HAZ.
Thiede & Strube, 2020 [5]	18 Sub-Saharan African countries	Panel study	Children under the age of 5	182,272	WHZ Wasting	Temperature anomalies (average temperature observed for a given cluster for the 12 months prior to the survey, standardized over all consecutive 12-month periods in the entire climate history for that location)	Two years	**WHZ:** an increase in temperatures from average to two standard deviations above average is associated with a 6.7% reduction in predicted WHZ, from 0.252 to 0.269 (p < 0.05). **Wasting:** the predicted probability of wasting increases by approximately 8.1 percent (from 9.9 to 10.7%) as temperatures increase by two standard deviations over the baseline average (p < 0.01).
Tusting et al., 2020 [36]	29 Sub-Saharan African countries	Panel study	Children under the age of 5	656,107	HAZ (stunting) WHZ (wasting) WAZ (underweight)	Monthly average daytime LST classified in three temperature bins: 30°C, 30°C to <35°C, and ≥35°C	Between 2000 and 2016	**Stunting**: odds of stunting were 10% lower among children living where the monthly average daytime LST exceeded 35°C, compared with those where monthly average daytime LST was less than 30°C (OR: 0.90, 95% CI 0.85–0.96). **Wasting:** the odds of wasting were 27% higher among children living where the monthly average daytime LST exceeded 35°C (OR: 1.27, 95% CI 1.16–1.38). **Underweight**: the odds of being underweight were 9% higher among children living where the monthly average daytime LST exceeded 35°C (OR: 1.09, 95% CI 1.02–1.16).
Wallwork et al., 2017 [41]	USA	Cohort study	Older men	587	Waist circumference (WC)	Average daily temperature	The year before each visit (2- to 5- year average)	**WC**: temperature was not significantly associated with both the risk of abdominal obesity (HR = 1.06, 95% CI: 0.86, 1.31 0.58) and metabolic syndrome as a whole (HR = 0.99, 95% CI: 0.82, 1.21)

Regarding study design, five were cohort studies and 15 were panel/cross-sectional studies. Geographically, the majority of the studies were developed in countries on the African continent (detailed in Table 2) (n = 11), with five of these being multi-country studies, and one was in Australia [n = 1], one in China [n = 1], two in the United States [n = 1], and four in South Asia. Another study was multi-country, but the countries involved were not individually identified (Table 2).

### Risk of bias

**Fig 2:**
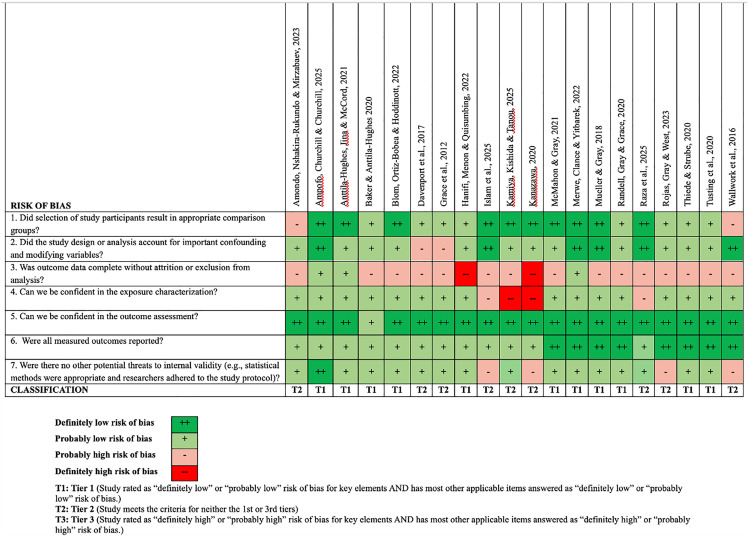
Summary of the risk of bias evaluation of cross-sectional and cohort studies using the OHAT instrument.

All the studies included in this systematic review were evaluated for risk of bias. Eleven publications (55%) were classified as Tier 1, meaning they presented either a probably or definitively low risk of bias for all the key criteria of the instrument, as well as for the majority of the other questions. Nine studies (37.5%) were classified as Tier 2, i.e., they did not present either a low or high risk of bias for all the key criteria (Fig 2). None of the studies were classified as Tier 3, that means the study presents a probably or definitively high risk of bias for all the key criteria and the majority of the other questions. The key questions of the instrument refer to: a) Is the exposure characterization trustworthy?; b) Is the outcome evaluation trustworthy?; and c) Did the study design or analysis take important confounding and modifying variables into consideration?

The main problems presented by the studies refer to incomplete outcome data, with a loss of participants, or exclusion in the analysis (n = 17), inappropriate statistical analysis (n = 04); confidence in relation to characterization of exposure (n = 4); inadequate selection of study participants (n = 02); and study design or analysis which did not take important confounding and modifying variables into consideration (n = 02). The majority of the studies were well classified in relation to characterization of exposure, outcome, and presentation of the results for all outcomes evaluated (Fig 2). No study was classified as Tier 3 (high risk of bias), and, in line with Cochrane [19] recommendations, none were excluded based on methodological quality.

### Exposure

The measurement of the ambient temperature varied considerably between studies, with some using distinct metrics within the same study. Most studies used mean temperature (daily, monthly, or annual) (n = 09), or maximum temperature (daily or monthly) (n = 4). One study used the El Nino 3.4 index (average monthly value of the index anomaly during the period between May and December); others adopted the concept of temperature anomalies, classified in distinct forms (n = 6); two used heatwaves, classified in different ways; and others adopted temperature categories (which also varied across studies) (n = 2). The temperature data were obtained from different sources, including weather forecasting centres, and air quality or meteorological monitoring stations (Table 2).

Considering the exposure window, the studies also showed considerable variation, even in the same study. Four of them assessed the effect of temperature on the anthropometric status of their populations, considering one year of exposure (the year prior to the interview) [14,27,34,41]. Three studies evaluated the effect of temperature from prenatal exposure up to the first or second year of life [4,33,35]. Five studies assessed the effect of temperature over lifetime period [8,28,30–32]. Two studies each considered the following exposure windows: temperature over the past 16 years [36,39] and temperature in the last two years [5,37]. One study each investigated the effect of temperature over the past five years [26], from the pre-natal to lifetime [38], from one year before birth to the year of the interview [29], and in one case, this information was unclear (possibly the year prior to the interview) [40] (Table 2).

### Outcome

The majority of the studies focused on children (<5 years old) evaluated the effects of high temperatures on the HAZ (n = 14), WHZ (n = 7), and WAZ (n = 4) anthropometric indicators (in a continuous and/or categorical form). For this population, the arm circumference (n = 1) and BMI-for-Age (BAZ) (n = 1) indicators were also evaluated. A study including adolescents and adults investigated the BMI [40]. The two studies conducted with adults and elderly people also used the BMI to assess the anthropometric status [14,39]. Lastly, a study conducted with elderly men evaluated the influence of high temperatures on the waist circumference [41] (Table 2).

### Associations between temperature and anthropometric indicators

#### Height-for-Age (HAZ).

The association between temperature and linear growth (HAZ/stunting) was explored in 13 studies, primarily centered in Sub-Saharan Africa (n = 10). While Table 2 provides a detailed study-by-study breakdown, the evidence reveals a complex landscape modulated by geographical and developmental contexts.

**Evidence of Adverse Impacts (n = 7):** A consistent detrimental effect of heat on HAZ was identified in seven studies [4,26,28,31,34,37,38]. In Pakistan and Ethiopia, temperature increases were linked to significant reductions in HAZ scores (β −0.24 [31] and −0.104 SD [4], respectively). On a larger scale, a 2ºC rise nearly doubled stunting prevalence in a five-country African analysis [28], while in Nigeria, a 1ºC increase raised stunting probability by 16.7%, particularly in rural areas [34]. Developmental timing emerged as a critical modifier: in Bangladesh, extreme heat during the postnatal window of the first 1,000 days (OR=1.67) was more damaging than in utero exposure (OR=1.28) [38]. However, regional variations exist; in Northern Mali, heat increased stunting odds (OR 1.52) [37], whereas in Uganda, heatwaves showed only a non-significant negative trend [26].

**Positive and Null Findings (n = 6):** Conversely, three studies reported positive associations [33,35,36] and three found no significant relationship [8,29,30]. Contextual factors likely explain these discrepancies. For instance, in Southern Mali, higher temperatures were associated with reduced odds of stunting [37], contrasting with the Northern region. In specific cohorts from Burkina Faso and other Asian/African countries, heat anomalies during early life unexpectedly improved HAZ [33,35], and high land surface temperatures (>35°C) were linked to reduced stunting odds in a 29-country analysis [36]. Meanwhile, large-scale studies in 30 Sub-Saharan and four South Asian countries found either null [8] or non-significant modest negative effects [29,30].

These divergent patterns suggest that the impact of thermal stress is not monolithic but is filtered through regional adaptive capacities, baseline climates, and potentially the protective or detrimental roles of local agricultural cycles.

#### Weight-for-Age (WAZ).

Five studies evaluated the impact of thermal stress on WAZ and underweight, with four identifying a consistent detrimental association [27,34,36,37] and one reporting null findings [8]. High-temperature exposure was significantly linked to increased underweight risk across different scales: in Mali, Kamiya et al. (2025) [37] found higher odds linked to average temperatures over the past year (OR = 1.57; p = 0.001) and two years (OR = 1.60; p = 0.002), while a 29-country analysis by Tusting et al. (2020) [36] showed that monthly surface temperatures exceeding 35ºC increased underweight chances by 9% (OR 1.09, 1.02–1.16; p = 0.0073) compared to temperatures under 30ºC. Similarly, Merwe et al. (2022) [34] observed that maximum monthly temperatures in Nigeria increased underweight prevalence, with results becoming more robust when adjusting for sociodemographic and regional characteristics. On a macro-climatic scale, Anttila-Hughes et al. (2021) [27] estimated that a 1ºC increase in the ENSO index was associated with a 0.03 SD reduction in WAZ (p = 0.02) and a 0.6% increase in underweight prevalence (p < 0.05). Conversely, Baker & Anttila-Hughes (2020) [8] did not identify any significant association between average temperatures and WAZ, suggesting that weight-based indicators may be more sensitive to extreme thresholds or specific climatic oscillations than to broad temperature averages.

#### Weight-for-Height (WHZ).

Seven studies investigated the influence of high temperatures on WHZ and wasting, with six identifying a detrimental association and one reporting no association [27]. Thermal stress showed a particularly acute impact on children under two in Pakistan, where a 1°C rise was linked to a significant decrease in WHZ scores (β = −0.33, 95% CI: −0.49 to −0.17) [31]. In Sub-Saharan Africa, the evidence consistently points to increased vulnerability: a 2ºC increase in monthly averages raised wasting prevalence from 4.1% to 6.2% [28], while annual temperature changes of 1ºC led to an 0.08 SD decline in WHZ [8]. Extreme thresholds were also critical, as surface temperatures over 35ºC were associated with 27% higher odds of wasting (OR=1.27; 95% CI = 1.16–1.38) [36].

Contextual and regional factors further modulated these effects. In Mali, the odds of wasting were exceptionally high in the northern region when average temperatures increased over the past year (OR = 5.668, p = 0.0001) and two years (OR = 2.654, p = 0.024) [37]. Similarly, across 16 African nations, a 2-SD increase above the average temperature resulted in a 6.7% reduction in the WHZ mean [5]. In contrast, Anttila-Hughes et al. (2021) did not identify a significant association between the ENSO index and wasting (p = 0.21) [27], suggesting that acute wasting may be more directly sensitive to local temperature extremes than to broader climatic oscillations.

#### Abdominal obesity.

Evidence regarding abdominal obesity was limited to a single study. Wallwork et al. (2017) [40] examined the long-term impact of average daily temperatures among the elderly, finding no statistically significant association with abdominal obesity (HR = 1.0; 95% CI: 0.86, 1.16; P = 1.00) or overall metabolic syndrome risk (HR = 0.99, 95% CI: 0.82, 1.21; P = 0.95), as detailed in Table 2.

#### Arm circumference.

A single study in Bangladesh [32] evaluated the impact of temperature variability during the month of birth on the nutritional status of children aged 0–3 years. The results indicated a dose-response detrimental effect on arm circumference: exposure to temperatures between 25°C and 30ºC was associated with a reduction in the indicator (β = −1.533; p < 0.01), while temperatures exceeding 30ºC resulted in an even more pronounced decline (β = −2.154; p < 0.01), as summarized in Table 2.

#### Body Mass Index (BMI).

Studies in adults and the elderly revealed distinct patterns of vulnerability based on age and climate. In the United States, Kanazawa (2020) [38] identified that maximum daily temperatures were positively associated with increased BMI (β = 0.036), weight (β = 0.098), and higher odds of being overweight (β = 0.008) or obese (β = 0.011; all p < 0.001). Similarly, Ampofo et al. (2025) [39] reported a causal link where each additional day of exposure to temperatures above 30°C per year was associated with a 0.02% increase in BMI (p = 0.02) and a 0.2% increase in obesity probability (p = 0.002).

In contrast, evidence from China [14] highlighted a shift toward underweight risk among older populations. Mueller & Gray (2018) found that temperature anomalies significantly increased the probability of being underweight, with the effect magnitude rising from the 41–60 age group (p = 0.003) to those over 60 (p = 0.004). Specifically, extremely hot and dry conditions were associated with a 3.3 percentage point increase in underweight status for the group aged over 60 [14] (Table 2).

### Meta-analysis

**Fig 3:**
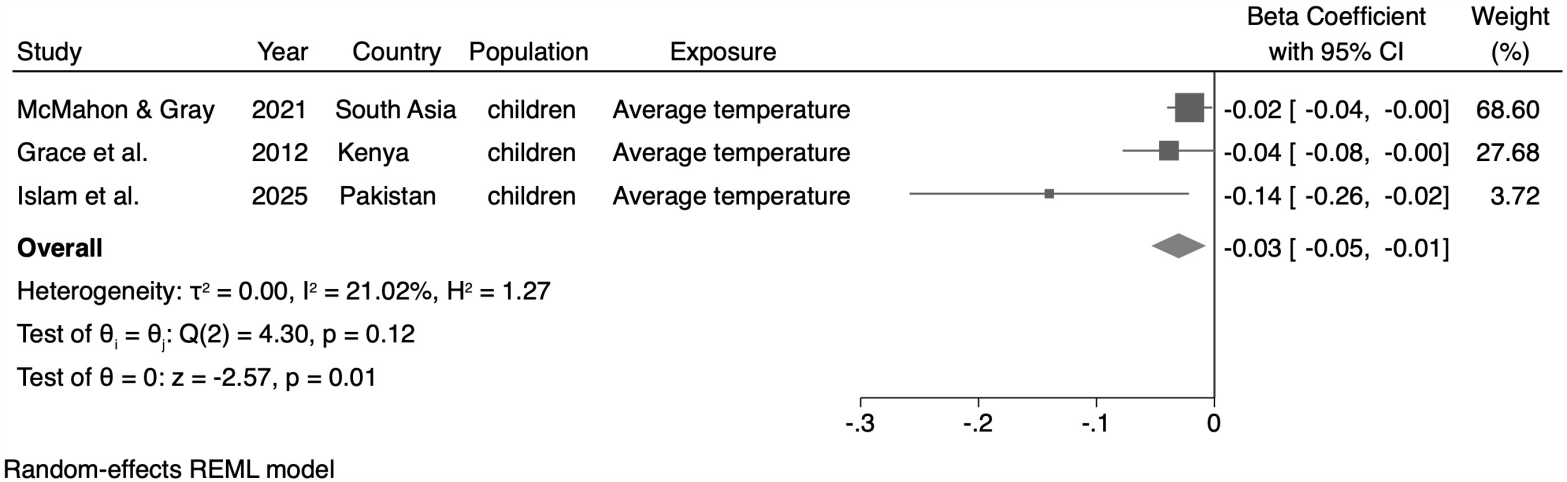
Meta-analysis of the association between the average temperature (monthly or yearly) and the height-for-age (HAZ) indicator.

**Fig 4:**
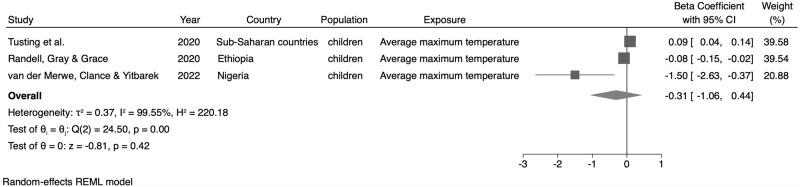
Meta-analysis of the association between maximum temperature (monthly or yearly) and the height-for-age (HAZ) indicator.

**Fig 5:**
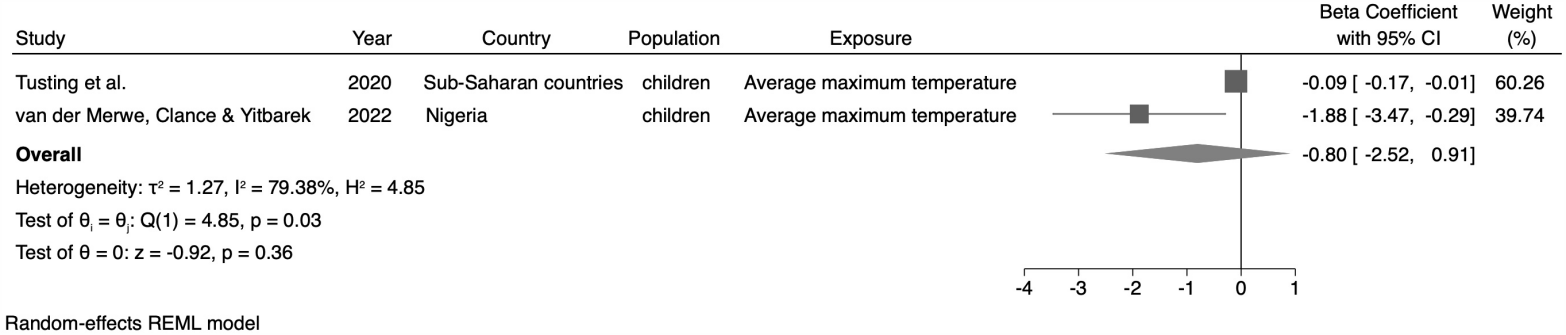
Meta-analysis of the association between maximum temperature (monthly or yearly) and the weight-for-age (WAZ).

**Fig 6:**
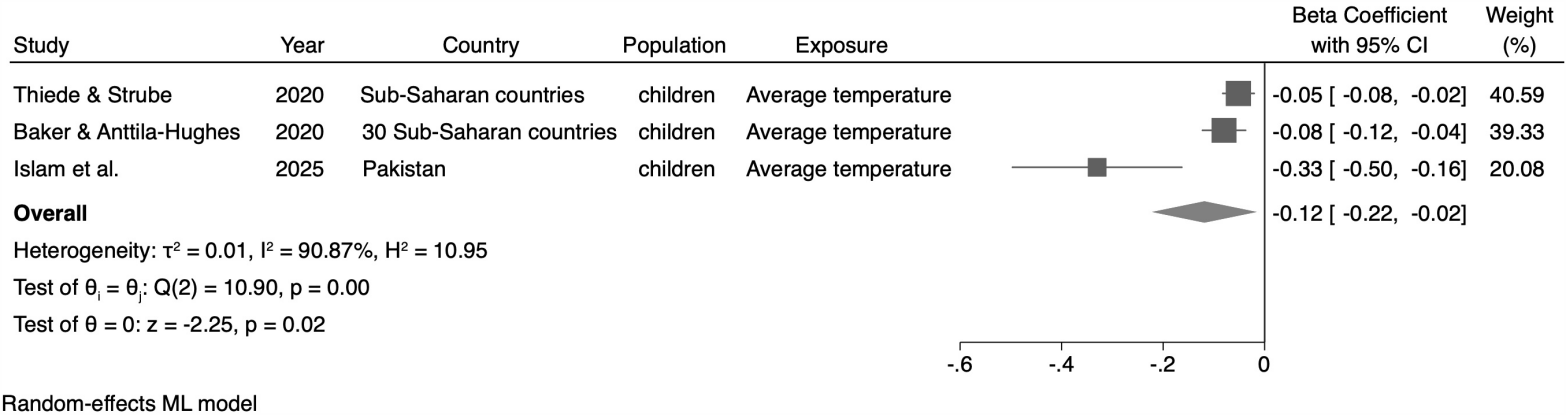
Meta-analysis of the association between average temperature (monthly or yearly) and the weight-for-height (WHZ) indicator.

The meta-analysis results are presented in Figs 3−6. We only conducted meta-analyses with studies that were combinable in terms of exposure (i.e., studies that used average temperature – monthly or yearly, or average maximum temperature -monthly or yearly), population (children younger than 5 years, of both sexes), and outcome (HAZ, WAZ, and WHZ indicators). When evaluating the effect of average temperature (monthly or yearly) on the HAZ indicator, we identified a 0.03σ reduction in the z-score average of this indicator for every 1ºC increase in average temperature (β = −0.03; 95% CI: −0.04; −0.01, n = 3 studies) (Fig 3). When analyzing the effect of the average maximum temperature (monthly or annual) on HAZ, no statistically significant association was found (β = −0.31; 95% CI: −1.06; 0.44, n = 3 studies) (Fig 4).

For the WAZ, no statistically significant association between the maximum average temperature and this indicator (β = −0.80; 95% CI: −2.52; 0.91, n = 2 studies) was identified (Fig 5). Regarding the WHZ, we observed a 0.12σ reduction in the z-score average for this indicator for every 1ºC increase in the average temperature (monthly or annual) (β = −0.12; 95% CI: −0.22; −0.02, n = 3 studies) (Fig 6).

The Grading of Recommendations, Assessment, Development and Evaluation (GRADE) approach was applied to the outcomes HAZ, WAZ, WHZ, BMI, WC, arm circumference, and BMI (Table 3). All outcomes started with low certainty, as they were based on observational studies. With the exception of arm circumference, all outcomes underwent at least one downgrade and were classified as having very low certainty, according to GRADE criteria. The domains with the most significant issues were inconsistency (substantial variability among results) and imprecision (uncertain effects and/or small sample sizes).

**Table 3:** Summary of Grading of Recommendations Assessment, Development, and Evaluation (GRADE).

No of Studies	Certainty assessment						Certainty
	Study design	Risk of bias	Inconsistency	Indirectness	Imprecision	Publication Bias	
**HAZ**							
13	Observational studies	Not serious	Serious^a^	Not serious	Not serious	Not serious	⨁⨁○○ Very low
**WAZ**							
05	Observational studies	Not serious	Serious^a^	Not serious	Not serious	Not serious	⨁⨁○○ Very low
**WHZ**							
07	Observational studies	Not serious	Moderate	Not serious	Not serious	Not serious	⨁⨁○○ Very low
**BMI**							
03	Observational studies	Not serious	Serious	Not serious	Serious	Not serious	⨁⨁⨁○ Very low
**WC**							
01	Observational studies	Not serious	Not serious	Not serious	Serious	Not serious	⨁⨁○○ Very low
**Arm Circumference**							
01	Observational studies	Not serious	Not serious	Not serious	Not serious	Not serious	⨁○○○ Low

a. Meta-analyses showed high heterogeneity.

## Discussion

This systematic review and meta-analysis provides evidence that exposure to high ambient temperatures is associated with adverse anthropometric outcomes, particularly in children under five years of age. Most studies originated from Sub-Saharan Africa and revealed mixed results for HAZ, but a consistent inverse association between high temperatures and WHZ/WAZ, indicating increased risk of wasting and underweight with rising temperatures. In contrast, studies among adults and older populations yielded more heterogeneous findings, ranging from weight loss to higher obesity prevalence. In the meta-analysis of combinable studies, we estimated a reduction in the Z-score average for the HAZ and WHZ indicators for every one degree increase in the average temperature (monthly or yearly). Although the results were statistically significant, the strength of these associations was weak, with a 0.03 reduction in the HAZ average and 0.12 reduction in the WHZ average. A significant association was not identified for the WAZ indicator.

Our review provides evidence of the effect of high ambient temperatures on both the classical measures of acute (WHZ) and chronic (HAZ) child malnutrition. We highlight that the mechanisms through which high temperatures affect chronic malnutrition may not be the same as those which affect acute malnutrition. Chronic and acute malnutrition are consequences of types of nutritional challenges. Chronic malnutrition reflects prolonged periods of insufficient nutrient intake, with permanent consequences for growth, while acute malnutrition reflects shorter periods of insufficient food intake from which children can recover if the food intake improves. For example, chronic malnutrition could be caused by long-term insufficient calorie intake, as a result of heat-induced crop failure. In contrast, acute malnutrition could result from heat-induced food spoilage, causing short-term illness [28].

Although the estimated effect sizes in the meta-analyses (e.g., −0.02 in HAZ and −0.06 in WHZ per 1 °C increase in average temperature) may appear modest at the individual level, their implications become substantial when viewed from a population perspective. Even small leftward shifts in Z-score distributions can translate into a considerable increase in the proportion of children crossing clinical thresholds for stunting or wasting – particularly in contexts where baseline prevalence is already high. Thus, while individual-level effects may seem minor, they are consistent, biologically plausible, and carry important public health implications.

The effect of temperature on the metabolic syndrome and abdominal obesity in the elderly was evaluated in one study only. Considering the vulnerability of elderly individuals, the need to evaluate the effect of temperature changes on malnutrition is relevant, since it is a frequent nutritional disorder in this population [42,43]. Only three studies were conducted to evaluate the effect of high temperatures on the BMI of adult individuals. One of these identified that temperature anomalies increase the probability of low weight [14], while a further two observed an increase in BMI for being overweight and obesity for every one degree increase in the maximum daily temperature [41] and a significant increase in obesity rates, with a one degree increase in the average annual temperature [39]. A number of authors suggest that increases in temperature may influence income [15], the price of food [16], preferences with food consumption [17] and levels of physical activity [18], particularly in low- and middle-income countries. In addition, changes in working hours, levels of physical activity, and minimum calorie requirements may also influence calorie intake. Any alteration in calorie intake and expenditure naturally has an impact on obesity. Obesity is one of the greatest global health challenges in recent decades. Thus, knowing the effects of the rising temperature increase on this condition is essential for policymakers and the general population.

We emphasize that in the 16 studies evaluated for risk of bias, the majority presented a good classification, and none were classified as Tier 3, which indicates a probably or definitively high risk of bias. We also highlight that the majority used Demographic and Health Survey (DHS) data, which covers large samples representative of the countries’ populations; they involved appropriate study designs to assess associations of interest, robust statistical analysis, accounting for confounding and effect modification, and the exposure and outcome measured appropriately. These results indicate the good quality of the studies that explore the association between high temperatures and the anthropometric status of the general population, making the findings of this review consistent and trustworthy. However, we should also highlight the great heterogeneity identified in the methods of measuring the exposure (temperature) in the different studies. Since there are no gold standards, each study adopted distinct measures, which made comparison difficult, indicating that the body of evidence is still limited and inconsistent for exposure. Thus, the development of studies which seek to define standards of temperature when used as an exposure is urgently required.

This systematic review and meta-analysis provide a comprehensive summary of the effects of high ambient temperature on malnutrition, including studies which use demographic and national health surveys from various countries. The inclusion of large sample sizes increases the accuracy, representativeness, and relevance of our findings. However, several limitations regarding geographical scope, demographic focus, and meteorological variables must be emphasized.

Firstly, most studies originated in Africa, with other tropical regions of South America and Asia being under-represented, limiting the applicability of findings to these regions. Another important limitation is that the majority of studies did not address issues of attribution and detection of causal pathways, which are essential to understanding the underlying mechanisms of this phenomenon. Furthermore, the disproportionate number of studies focusing on pediatric populations represents a significant constraint. This demographic imbalance restricts the generalizability of our findings to other vulnerable groups and life stages, such as pregnant women, middle-aged adults, and the elderly, whose physiological demands and thermoregulatory responses to thermal stress differ substantially from those observed in children.

In addition, we must acknowledge the substantial methodological heterogeneity observed in exposure assessment. In the absence of a universal “gold standard” for environmental thermal stress, researchers have adopted a wide array of metrics and diverse exposure definitions (e.g., varying thresholds for heatwaves and temperature anomalies). This lack of standardization, combined with the small number of studies contributing to specific meta-analyses, prevented the performance of more robust subgroup analyses as originally intended in the protocol. These factors complicate the cross-comparison of effect sizes and suggest that the current body of evidence remains fragmented. Consequently, the development of standardized exposure protocols and the harmonization of temperature metrics are urgently required to strengthen the evidence base for climate-health policy. Finally, the limited comparability of studies regarding key population characteristics, such as the inability to stratify by age or sex in most cases, may have contributed to the modest effect sizes observed, given that nutritional vulnerability and growth trajectories differ substantially across these groups.

However, despite the aforementioned limitations, the meta-analysis conducted here offers preliminary evidence of a potential association between temperature and nutrition, indicating that elevated temperatures could adversely influence growth and nutritional status under certain conditions. While it remains premature to draw definitive policy conclusions, these findings underscore the importance of further research to better characterize these associations. Such efforts are essential to eventually inform the development of context-specific public health and climate adaptation strategies, particularly in tropical and low- and middle-income countries where socioeconomic and environmental vulnerability may be greatest.

Future studies are also needed to better assess the effects of elevated ambient temperature on nutritional outcomes in the general population. The observed inconsistencies in HAZ outcomes, combined with limitations related to age range, geographic distribution, and high heterogeneity in temperature estimation methods, reveal significant gaps in the current body of evidence. Both short- and long-term effects should be investigated across different contexts and population groups, including the identification of potential mediators and effect modifiers of this association, such as droughts and heavy rainfall, to better understand the pathways of these relationships.

## Conclusion

The findings of this review underscore the need for further research on the potential effects of high ambient temperatures on anthropometric indicators. Although the meta-analyses detected modest reductions in HAZ and WHZ Z-scores per 1°C increase, these results should be interpreted cautiously due to the low certainty of evidence, inconsistencies across studies, and substantial heterogeneity in exposure assessment, age groups, and geographic coverage. Current evidence is insufficient to draw definitive conclusions about causal pathways or direct policy implications. Nonetheless, the observed patterns suggest a potential adverse impact of elevated temperatures on nutritional status, highlighting the importance of continued investigation. Future longitudinal, high-quality, and geographically diverse studies are critical to clarify these associations and inform public health strategies. Strengthening the evidence base will be essential to guide targeted interventions and climate adaptation policies aimed at mitigating the nutritional consequences of rising temperatures.

## Supporting information

S1 Table PRISMA checklist. (DOCX)

S2 Table Search Strategy for each Database. (DOCX)

S3 File Database file. (XLSX)
