# Supplementary material for: High ambient temperature effects on the anthropometric status of the population: A systematic review and meta-analysis
Source: PLoS One. 2026 Apr 1;21(4):e0344186. doi: 10.1371/journal.pone.0344186 (PMC13042653; doi:10.1371/journal.pone.0344186)
Supplement: S2 Table — (DOCX) [file pone.0344186.s002.docx]

**S2 Table. Search Strategy for each Database**

| **Database** | **Strategy** |
| --- | --- |
| PUBMED | **#1** (((((((((((((((((((((((Adiposity[MeSH Terms]) OR (Body Fat Patterning[MeSH Terms])) OR (Body Compositions[MeSH Terms])) OR (Subcutaneous Fat, Abdominal[MeSH Terms])) OR (Intra-Abdominal Fat[MeSH Terms])) OR (Abdominal Fats[MeSH Terms])) OR (Visceral Obesity[MeSH Terms])) OR (Starvation[MeSH Terms])) OR (Severe Acute Malnutrition[MeSH Terms])) OR (Malnutrition[MeSH Terms])) OR (Deficiency Diseases[MeSH Terms])) OR (Nutrition Disorders[MeSH Terms])) OR (Nutritional and Metabolic Diseases[MeSH Terms])) OR (Obesity, Severe[MeSH Terms])) OR (Obesity[MeSH Terms])) OR (Excess weight[MeSH Terms])) OR (Overweight[MeSH Terms])) OR (Waist-Height Ratio[MeSH Terms])) OR (Waist Circumference[MeSH Terms])) OR (Body Weight[MeSH Terms])) OR (Body Height[MeSH Terms])) OR (Body Mass Index[MeSH Terms])) OR (Anthropometry[MeSH Terms])) OR (Nutrition Status[MeSH Terms]))  **#2** Climate[MeSH Terms] OR Climate Change[MeSH Terms] OR Tropical Climate[MeSH Terms] OR Desert Climate [MeSH Terms] OR (Heatwaves) OR (Heat waves)) OR (Global warming[MeSH Terms])) OR (Extreme Weather[MeSH Terms])) OR (Heat[MeSH Terms])) OR (Hot Temperature[MeSH Terms])) OR (Hot Weather[MeSH Terms])) OR (Extreme Hot Weather[MeSH Terms])) OR (extreme heat[MeSH Terms])) OR (high temperature)  **#3** child[MeSH Terms] OR infant[MeSH Terms] OR infant, newborn[MeSH Terms] OR adolescent[MeSH Terms] OR adult[MeSH Terms] OR aged[MeSH Terms] OR elderly OR pregnant[All Fields] |
| EMBASE | **#1** 'obesity'/exp OR ‘abdominal subcutaneous fat’/exp OR ‘intra-abdominal fat’/exp OR ‘starvation’/exp OR ‘malnutrition’/exp OR ‘nutritional deficiency’/exp OR ‘nutritional disorder’/exp OR ‘nutritional and metabolic disorder’/exp OR ‘waist to height ratio’/exp OR ‘waist circumference’/exp OR ‘body weight’/exp OR ‘body height’/exp OR ‘body mass’/exp OR ‘anthropometry’ OR ‘nutritional status’/exp  **#2** ‘heat wave’/exp OR ‘greenhouse effect’/exp OR ‘extreme weather’/exp OR ‘heat’/exp OR ‘high temperature’/exp OR ‘extreme hot weather’/exp  **#3** 'children'/exp OR 'child'/exp OR 'teenager'/exp OR 'adolescent'/exp OR 'adults'/exp OR 'grown-ups'/exp OR 'grownup'/exp OR 'grownups'/exp OR 'adult'/exp OR 'aged people'/exp OR 'aged person'/exp OR 'aged subject'/exp OR 'elderly'/exp OR 'elderly people'/exp OR 'elderly person'/exp OR 'elderly subject'/exp OR 'senior citizen'/exp OR 'aged'/exp OR 'infant'/exp OR 'child, newborn'/exp OR 'neonate'/exp OR 'newborn child'/exp OR 'pregnant women'/exp OR 'pregnant woman'/exp |
| BVS  AND ( db:("LILACS" OR "IBECS" OR "CUMED" OR "BINACIS" OR "BDENF" OR "LIPECS" OR "ARGMSAL" OR "VETINDEX")) | **ENGLISH**  **#1** *(Adiposity ) OR (Overweight ) OR (Fat Patterning ) OR (Body Composition ) OR (Subcutaneous Fat, Abdominal) OR (Intra-Abdominal Fat ) OR (Abdominal Fats ) OR (Abdominal Adipose Tissue ) OR (Abdominal Fats ) OR (Adipose Tissue, Abdominal ) OR (Fat, Abdominal ) OR (Fats, Abdominal ) OR (Visceral Obesity ) OR (Starvation) OR (Severe Acute Malnutrition ) OR (Malnutrition) OR (Deficiency Diseases ) OR (Nutrition Disorders ) OR (Nutritional and Metabolic Diseases ) OR (Obesity, Severe ) OR (Obesity) OR (Waist-Height Ratio ) OR (Waist Circumference ) OR (Body Weight ) OR (Body Height) OR (Body Mass Index ) OR (Anthropometry) OR (Nutrition Status )*  **#2** *(Heatwaves ) OR (Heat waves ) OR (Global warming ) OR (Extreme Weather ) OR (Heat ) OR (Hot Temperature ) OR (Hot Weather ) OR (Extreme Hot Weather) OR (extreme heat) OR (High Temperature)*  **#3** (children) OR (child) OR (Infant, Newborn) OR (Infants, Newborn) OR (Neonate) OR (Neonates) OR (Newborn) OR (Newborn Infant) OR (Newborn Infants) OR (Newborns) OR (infant) OR (infants) OR (teenager) OR (teenagers) OR (adolescent) OR (Adolescence) OR (Adolescents) OR (Teen) OR (Teens) OR (Youth) OR (Youths) OR (adults) OR (adult) OR (aged) OR (elderly) OR (pregnant women) OR (pregnant woman) OR (woman pregnant) OR (women pregnant)  **SPANISH**  **#1** Adiposidad OR Distribución de la Grasa Corporal OR Composición Corporal OR Grasa Subcutánea Abdominal OR Grasa Abdominal OR Grasa Intraabdominal OR Tejido Adiposo Abdominal OR Obesidad Abdominal OR Inanición OR Desnutrición Aguda Severa OR Desnutrición OR Enfermedades Carenciales OR Trastornos Nutricionales OR Enfermedades Nutricionales y Metabólicas OR Obesidad Mórbida OR Obesidad OR Sobrepeso OR Relación Cintura-Estatura OR Circunferencia de la Cintura OR Peso Corporal OR Estatura OR Índice de Masa Corporal OR Índice de Quetelet OR Antropometría OR Estado Nutricional  **#2** Ola de Calor OR Rayos Infrarrojos OR Calentamiento Global OR Clima Extremo OR Calor Extremo OR Clima Calido Extremo OR Clima Cálido Extremo OR Calor OR Temperatura alta OR Temperatura elevada  **#3** Niño OR Niños OR Lactante OR Lactantes OR Recién Nacido OR Lactante Recién Nacido OR Lactantes Recién Nacidos OR Neonato OR Neonatos OR Niño Recién Nacido OR Niños Recién Nacidos OR Recién Nacidos OR Adolescente OR Adolescencia OR Adolescentes OR Joven OR Jóvenes OR Juventud OR Anciano OR Adulto Mayor OR Ancianos OR Persona de Edad OR Persona Mayor OR Personas de Edad OR Personas Mayores OR Mujeres Embarazadas OR Embarazadas OR Mujer Embarazada  **FRENCH**  **#1** Adiposité OR Répartition du tissu adipeux OR Composición Corporal OR Graisse sous-cutanée abdominale OR Graisse intra-abdominale OR Graisse abdominale OR Tissu adipeux abdominal OR Tissu graisseux abdominal OR Obésité abdominale OR Inanition OR Malnutrition aigüe sévère OR Malnutrition OR Maladies de carence OR Troubles nutritionnels OR Troubles de la nutrition OR Maladies métaboliques et nutritionnelles OR Obésité morbide OR Obésité OR Surpoids OR Excès de poids OR Excès ponderal OR Surcharge pondérale OR Surplus de poids OR Rapport tour de taille sur taille OR Tour de taille OR Poids OR Taille OR Indice de masse corporelle OR IMC (Indice de Masse Corporelle) OR Règle de Quetelet OR Règle de Quételet OR Anthropométrie OR État nutritionnel  **#2** Vague de Chaleur OR Réchauffement de la planète OR Conditions météorologiques exceptionnelles OR Chaleur extreme OR Temps extrêmement chaud OR Très grande chaleur OR Température élevée  **#3** Enfant OR Nourrisson OR Bébé OR Enfant en bas âge OR Nouveau-né OR Adolescent OR Adulte OR Sujet âgé OR Adulte âgé OR Personne âgée OR Personne du troisième âge OR Femmes enceintes |
| WEB OF SCIENCE | **#1 “**Adiposity” OR “Body Fat Patterning” OR “Body Compositions” OR “Subcutaneous Fat, Abdominal” OR “Intra-Abdominal Fat” OR “Abdominal Fats” OR “Visceral Obesity” OR “Starvation” OR “Severe Acute Malnutrition” OR “Malnutrition” OR “Deficiency Diseases” OR “Nutrition Disorders” OR “Nutritional and Metabolic Diseases” OR “Obesity, Severe” OR “Obesity” OR “Excess weight” OR “Overweight” OR “Waist-Height Ratio” OR “Waist Circumference” OR “Body Weight” OR “Body Height” OR “Body Mass Index” OR “Anthropometry” OR “Nutrition Status”  **#2** “Heatwaves” OR “Heat waves” OR “Infrared Rays” OR “Global warming” OR “Extreme Weather” OR “Heat” OR “Hot Temperature” OR “Hot Weather” OR “Extreme Hot Weather” OR “Extreme heat” OR “High Temperature” OR “Heat”  **#3** "children" OR "child" OR “Infant, Newborn” OR “Infants, Newborn” OR “Neonate” OR “Neonates” OR “Newborn” OR “Newborn Infant” OR “Newborn Infants” OR “Newborns” OR “infant” OR “infants” OR “teenager” OR “teenagers” OR “adolescent” OR “Adolescence” OR “Adolescents” OR “Teen” OR “Teens” OR “Youth” OR “Youths” OR “adults” OR “adult” OR “aged” OR “elderly” OR “pregnant women” OR “pregnant woman” OR “woman pregnant” OR “women pregnant” |
| GOOGLE SCHOLAR | **#1** “Adiposity” OR “Body Fat Patterning” OR “Body Compositions” OR “Subcutaneous Fat, Abdominal” OR “Intra-Abdominal Fat” OR “Abdominal Fats” OR “Visceral Obesity” OR “Starvation” OR “Severe Acute Malnutrition” OR “Malnutrition” OR “Deficiency Diseases” OR “Nutrition Disorders” OR “Nutritional and Metabolic Diseases” OR “Obesity, Severe” OR “Obesity” OR “Excess weight” OR “Overweight” OR “Waist-Height Ratio” OR “Waist Circumference” OR “Body Weight” OR “Body Height” OR “Body Mass Index” OR “Anthropometry” OR “Nutrition Status”  AND  **#2** “Heatwaves” OR “Heat waves” OR “Infrared Rays” OR “Global warming” OR “Extreme Weather” OR “Heat” OR “Hot Temperature” OR “Hot Weather” OR “Extreme Hot Weather” OR “Extreme heat” OR “High Temperature” OR “Heat”  AND  "children" OR "child" OR “Infant, Newborn” OR “Infants, Newborn” OR “Neonate” OR “Neonates” OR “Newborn” OR “Newborn Infant” OR “Newborn Infants” OR “Newborns” OR “infant” OR “infants” OR “teenager” OR “teenagers” OR “adolescent” OR “Adolescence” OR “Adolescents” OR “Teen” OR “Teens” OR “Youth” OR “Youths” OR “adults” OR “adult” OR “aged” OR “elderly” OR “pregnant women” OR “pregnant woman” OR “woman pregnant” OR “women pregnant” |
